# Exosomal miR-500 Derived From Lipopolysaccharide-Treated Macrophage Accelerates Liver Fibrosis by Suppressing MFN2

**DOI:** 10.3389/fcell.2021.716209

**Published:** 2021-10-05

**Authors:** Lisha Chen, Yan Huang, Zhixi Duan, Peiqi Huang, Hongbing Yao, Yu Zhou, Qin Ji, Xiangfeng Liu

**Affiliations:** ^1^Central Laboratory, Department of Neurosurgery, The Second Xiangya Hospital, Central South University, Changsha, China; ^2^Affiliated Changsha Hospital of Hunan Normal University, The Fourth Hospital of Changsha, Institute of Emergency and Critical Care Medicine of Changsha, Changsha, China; ^3^Department of Emergency Medicine, Trauma Center, The Second Xiangya Hospital, Central South University, Changsha, China; ^4^Department of Hepatobiliary and Pancreatic Surgery, The Second Affiliated Hospital, Guilin Medical University, Guilin, China

**Keywords:** liver fibrosis, hepatic stellate cell, macrophage, exosome, miRNA

## Abstract

Liver fibrosis is an outcome of chronic hepatic injury, which can eventually result in cirrhosis, liver failure, and even liver cancer. The activation of hepatic stellate cell (HSC) is a prominent driver of liver fibrosis. Recently, it has been found that the crosstalk between HSCs and immune cells, including hepatic macrophages, plays an important role in the initiation and development of liver fibrosis. As a vital vehicle of intercellular communication, exosomes transfer specific cargos into HSCs from macrophages. Here, we show that exosomes derived from lipopolysaccharide (LPS)-treated macrophages has higher expression level of miR-500. And overexpression or inhibition of miR-500 in macrophage exosomes could promote or suppress HSC proliferation and activation. Treatment of exosomes with miR-500 overexpression can accelerate liver fibrosis in CCl_4_-induced liver fibrosis mouse model. miR-500 promotes HSC activation and liver fibrosis *via* suppressing MFN2. Moreover, miR-500 in serum exosomes could be a biomarker for liver fibrosis. Taken together, exosomal miR-500 derived from LPS-activated macrophages promotes HSC proliferation and activation by targeting MFN2 in liver fibrosis.

## Introduction

Liver fibrosis is a common pathological outcome of chronic hepatic disease (CHD), which can easily develop into cirrhosis or hepatocellular carcinoma, furtherly leading into the death (Parsons et al., 2007; Friedman, 2008; Hernandez-Gea and Friedman, 2011; Parola and Pinzani, 2019). The etiology of liver fibrosis includes viral infection, alcohol abuse, metabolic and autoimmune disease, etc. (Parsons et al., 2007; Friedman, 2008; Hernandez-Gea and Friedman, 2011). Despite the improved understanding of pathogenesis of liver fibrosis during the past decades, the detailed mechanisms are still needed to be clarified.

Liver fibrosis is characterized as accumulation of extracellular matrix (ECM) such as α-smooth muscle actin (α-SMA) in the liver, consequently contributing to liver fibrosis and later cirrhosis (Bataller and Brenner, 2005). Hepatic stellate cell (HSC) is the primary driver of liver fibrosis. Activated HSCs will develop into fibrogenic myofibroblast−like cells (activated HSCs) that secretes α−SMA, TIMP−1, and Collagen I (Yin et al., 2013; Wallace et al., 2015). Besides HSCs, immune cells, such as T and B lymphocytes, NK cells and macrophages, are also important player in the process of liver fibrosis (Casini et al., 1985; Bhogal and Bona, 2005; Ramachandran and Iredale, 2012; Glassner et al., 2013; Liu et al., 2019). Among them, macrophages play an essential role in the liver inflammation and the pathogenesis of liver injury and repair (Heymann et al., 2009). Macrophages, an important component in innate immune responses, are the first line of host defense against external infection or internal injury (Heymann et al., 2009; Ramachandran and Iredale, 2012; Pradere et al., 2013; Li et al., 2016). A broad array of sensing molecules is expressed on the membrane of macrophages, allowing them to monitor the microenvironment and resist against infection. Liver macrophages are composed of resident hepatic macrophages, named as Kupffer cells, and recruited bone marrow-derived macrophages (BMDMs), which migrate and infiltrate into the liver under the pathologic conditions (Tacke and Zimmermann, 2014). The cells are typically categorized into proinflammatory or “M1” and anti-inflammatory or “M2” phenotype. The proinflammatory macrophages are induced mainly by IFN-γ and lipopolysaccharide (LPS), while the anti-inflammatory macrophages are induced by Th2 cytokines such as IL-4 (Lawrence and Natoli, 2011). Bacterial LPS, is a well-known inducer of inflammation, has been demonstrated to be involved with hepatic fibrogenesis (Fouts et al., 2012). It may induce the macrophages to develop into M1 type, which produces and releases some cytokines and chemicals, such as TGF-β1 (TGF-β) and IL-6, thereby contributing to HSC activation and liver fibrosis (Fouts et al., 2012).

Accumulating evidence has shown that the crosstalk between macrophages and HSCs might have a direct influence on the outcome of hepatic injury. In brief, Kupffer cells are activated in CHD, and circulating macrophages are also recruited. The two groups of cells can secrete pro-inflammatory cytokines and chemokines, consequently resulting in the infiltration of other immune cells into liver tissues as well as hepatocyte apoptosis and HSC activation. Kupffer cells also can activate HSCs *via* paracrine mechanisms, involving the production and secretion of the profibrotic factors TGF-β and PDGF (Ramachandran and Iredale, 2012; Li et al., 2016). Actually, other types of communication pathway may also exist, which are needed for further study.

In recent years, it has been found that exosome secretion is one most common alternative communication pathway between cells and it has been reported to be participated in many biological and pathological processes (Thery et al., 2002; Johnstone, 2006; van Niel et al., 2006). Exosomes are small (30–150 nm) extracellular membrane vesicles, which contain proteins and nucleic acids including mRNAs, microRNAs (miRNAs), and long non-coding RNAs (lncRNAs). They are released into traverse intercellular spaces from donor cells, and then taken up by recipient cells. Being a vehicle, exosomes play an important role in cell communication. They can deliver proteins and nucleic acids, including miRNAs, lncRNAs, and circular RNA (circRNAs), consequently regulating intracellular signaling pathways in recipient cells (Thery et al., 2002; Johnstone, 2006; van Niel et al., 2006). miRNAs are a group of non-coding RNA, which have been identified to be involved in many diseases, *via* binding with 3′ UTR of their target genes and regulating their expression (Bartel, 2004). miRNAs are also involved in the progression of liver fibrosis. For instance, miR-34a-5p could inhibit liver fibrosis development by affecting TGF-β/Smad3 pathway in HSCs (Feili et al., 2018); miR-98 inhibits HSC activation, consequently attenuating the development of liver fibrosis (Wang Q. et al., 2020); miR-29a and miR-652 prevent liver fibrosis by suppressing CD4+ T cell differentiation (Xuan et al., 2017); TGF-β can induce liver fibrosis by downregulation of augmenter of liver regeneration in HSCs that is mediated by miR-181a (Gupta et al., 2019). Recent studies have revealed that exosomes-mediated shuttle of miRNAs is involved in the process of liver fibrosis. For instance, exosomal miR-223 derived from natural killer cells can inhibit HSC activation by suppressing autophagy (Wang L. et al., 2020); exosomes derived from miR-181-5p-modified adipose-derived mesenchymal stem cells attenuated liver fibrosis by activating autophagy (Qu et al., 2017); exosomal miR-214 from HSCs could regulate CCN2 expression in primary mouse hepatocytes (Chen et al., 2014); furthermore, fibrogenic signaling is also suppressed in HSCs through CCN2 regulating by exosomal miRNA-199a-5p (Chen et al., 2016); in our previous study, we found that exosomes derived from LPS-activated macrophages could be taken up by neighbor HSCs, consequently transferring miR-103-3p to these HSCs. miR-103-3p could promote HSC activation by suppressing Krüppel-like factor 4 (KLF4) (Chen et al., 2020).

In this going on work, we aimed to explore the effects of exosomal miR-500 derived from LPS-activated macrophages on HSCs. We investigate the influence of exosomal miR-500 on HSC proliferation and activation and the effect of miR-500 to accelerate the accumulation of EMC *in vitro* and *in vivo*. We also determined that the effect of miR-500 was associated with its targeting gene MFN2, subsequently suppressing TGF−β/Smad pathway. In addition, miR-500 in serum exosomes from patients in S1–S4 stages was increased compared with that in S0 stage. Therefore, circulating exosomes miR-500 could be function as a potential biomarker for the diagnosis of advanced liver fibrosis.

## Reagents and Methods

### Liver Tissues and Serum From Chronic Hepatic Disease Patients

The liver tissue and serum samples were obtained from patients with CHD. A total of 30 human liver tissues, including 6 fibrotic stage S0 (non-fibrosis), 6 S1 (mild fibrosis), 6 S2 (moderate fibrosis), 6 S3 (clear fibrosis), and 6 S4 (cirrhosis), were included in this study. The fibrotic stage was determined in accordance with the Scheuer’s classification. The written informed consent have been provided, and the study was approved by the Ethics Committee of the Second Xiangya Hospital of Central South University.

### Cell Culture

The mouse macrophage cell line (RAW264.7) was cultured in DMEM (Gibco) supplemented with 10% fetal bovine serum (FBS, Gibco) and 1% penicillin–streptomycin. To activate RAW264.7, the cells were stimulated with 100 ng/ml of LPS in the absence of FBS and antibiotics for 12 h. And then, exosomes were extracted from the supernatant. In addition, primary mouse HSCs were isolated from healthy male C57BL/6 (C57/B6) mice (6–8 weeks), as previously described by buoyant-density centrifugation. The primary mouse HSCs were cultured with fresh DMEM, F12, and 10% FBS medium. The cells were split 1:4 every 3 days and can be used at passages 0–6 (P0–P6), when they are considered as non-activated HSCs. HEK 293 cells were cultured in DMEM with 10% FBS.

### Cell Transfection

miR-500 mimic, miR-500 inhibitor, negative control (NC) mimic, and NC inhibitor were purchased from GenePharma (Shanghai, China). The MFN2 overexpression vector (pcDNA-MFN2), and relative control vector were obtained from Genecopoeia (Guangzhou, China). Cell transfection was performed by using the Lipofectamine 3000 reagent in accordance with the manufacturer’s protocal (Invitrogen). The AVV package (AVV-control and AVV-MFN2) were obtained from GenePharma.

### Exosome Isolation and Quantification

Exosomes were isolated at 100,000 × *g* ultracentrifugation of macrophage supernatant (following at least 1:4 dilution) for at least 18 h in accordance with the 2018 MISEV guideline. The morphological characteristics were identified by transmission electron microscopy (TEM) as described previously. Western blot analysis was applied for detecting the protein levels of exosome markers, including CD9 and CD81 (Systemic Bioscience).

### Cell Treatment

The HSCs was treated with 40 μg exosomes obtained from LPS-activated macrophages or control cells for 48 h at 37°C. After that, HSCs were harvested for further studies.

### qRT-PCR

The RNA was extracted by using TRIzol reagents (Thermo Fisher), and reversely transcribed to cDNA by reverse reaction kit following the manufacturer’s instruction (Promega). The quantitative real-time PCR analysis was conducted by using SYBR Green Master mix. GAPDH was determined to be as an internal gene, and the relative mRNA level of target gene was normalized to GAPDH by using 2^–ΔΔCT^ method. The MFN2 primer sequences for qPCR used in the study were F: CATTGCTGACAGGATGCAGAAGG R: TGCTGGAAGGTGGACAGT GAGG. The detection for miRNAs was conducted by miRNA Detection kit (GenePharma). The expression level of the miRNA was normalized to the relative expression of U6.

### Cell Proliferation and Cell Cycle Assay

To determine HSC proliferation, CCK-8 assays were applied to determine the cell proliferation. CCK-8 The cells were seeded into a 96-well plate, and then treated with exosomes or transfected with miR-500 mimcs or inhibitor. Then, the CCK-8 reagent was added per well and incubated for 1.5 h. We performed this assay at 0, 24, 48, and 72 h. The cell cycle of HSCs was analyzed using flow cytometry.

### Luciferase Activity Assay

The 3′ UTR fragment of MFN2 (wild type, WT) that contains the possible sites binding with miR-500 was synthesized and cloned into the pMIR-REPORT vector to generate luciferase reporter constructs. The mutant plasmid (MUT) was generated. For detecting the luciferase activity, these WT or MUT plasmid was transfected into HEK293 cells together with miR-500 mimic by using lipofectamine 3000 transfection reagent. Meanwhile, the pMIR-REPORT-β-gal vector was used as control. The relative luciferase activity was determined by using a luciferase reporter assay system (Promega).

### Western Blot

The exosomes and treated cells were lysed, and then centrifuged at 12,000 rpm for 15 min at 4°C. The protein concentration was detected by using a Pierce BCA Protein Assay kit (Thermo Fisher). The protein was electrophoresed by SDS-PAGE and then transferred to the PVDF membranes. The membranes were incubated with the primary antibodies, including anti−MFN2, α-SMA, TGF−β, Smad2/p−Smad2, and Smad3/p−Smad3 (Cell Signaling Technology) at 4°C overnight. Next, the blots were incubated with secondary antibodies. GAPDH was used as a loading control.

### CCl_4_−Induced Liver Fibrosis Mouse Model

C57/B6 mice (male) were purchased from Vital River Laboratory. The mice were administrated with CCl_4_ (5% CCl_4_ in olive oil; Sigma-Aldrich) twice a week for 4 weeks. Control groups were only treated with olive. Forty micrograms exosomes with miR-500 overexpression or not were administered through tail vein injection twice each week for 8 weeks. Furthermore, in some experiments, 2.5 × 10^11^ vg/mouse AAV−Control or AAV−MFN2 was also administrated through intravenous injection twice a week after the first CCl_4_ administration. On death, animals were euthanized by CO*2* inhalation. And then liver tissues and serum were collected for further studies. All the animal experiments in this study were approved by the Institutional Animal Care and Use Committee of the Second Xiangya Hospital of Central South University.

### Hematoxylin–Eosin, Masson, and Sirius Red Staining

The liver sections were stained with hematoxylin–eosin (H&E), Masson, or Sirius Red for histopathological examination. In brief, liver tissues were fixed and embedded in paraffin. The tissue section was deparaffinized and stained with H&E, Masson, or Sirius Red to evaluate the morphological changes and liver fibrosis.

### Serum Alanine Aminotransferase and Aspartate Aminotransferase

Serum samples from mouse were collected and serum alanine aminotransferase (ALT) and aspartate aminotransferase (AST) was assessed with a commercial kit (Rongsheng, Shanghai, China).

### Statistical Analysis

All statistical analyses were performed by SPSS 15.0. All the data are expressed as mean ± SD. The results were analyzed by ANOVA followed by the *post hoc* Dunnett’s test for multiple comparisons. *P* < 0.05 was considered statistically significant.

## Results

### Exosomal miR-500 Was Upregulated in Liver Fibrosis

**FIGURE 1:**
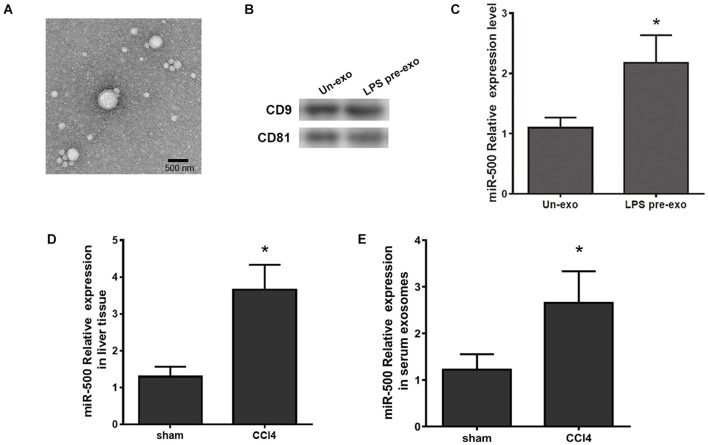
Exosomes derived from LPS-activated macrophages have higher expression level of miR-500. **(A)** Exosomes were isolated from LPS-pretreated macrophages (LPS pre-exo) and non-pretreated macrophages (un-exo), and then visualized by TEM. The representative image is shown. **(B)** The protein level of CD9 and CD81 in exosomes were detected by western blotting assays. **(C)** The expression levels of miR-500 in exosomes isolated from LPS pre-exo and un-exo were detected by real time PCR. **(D)** The expression levels of miR-500 in CCl_4_-induced liver fibrosis mice were detected by real time PCR. **(E)** The expression levels of miR-500 in serum exosomes isolated from CCl_4_-induced liver fibrosis mice and control mice were detected by real time PCR. These results are mean ± SD of three independent experiments. *P < 0.05.

In our previous study, we identified a series of dysregulated exosomal miRNAs in LPS-stimulated THP-1 macrophages and found the effects of some exosomal miRNAs on HSC activation in liver fibrosis. Herein, we aimed to confirm the functions of exosomal miR-500 in the communication between macrophages and HSCs. Firstly, we extracted exosomes from LPS-treated RAW264.7 and control cells and then they were identified by TEM (Figure 1A). The protein markers for exosomes, CD9 and CD81, were also detected by western blot (Figure 1B). miR-500 was significantly increased in exosomes that were derived from LPS-treated macrophages compared with non-treated cells (Figure 1C). miR-500 expression was significantly upregulated in liver in CCl_4_-induced liver fibrosis mouse model (Figure 1D). And the serum exosomal miR-500 was also increased in CCl_4_-induced liver fibrosis (Figure 1E). These results showed a changed level of exosomal miR-500 in liver fibrosis, which indicated that exosomal miR-500 might be involved in the process of liver fibrosis.

### miR-500 and Exosomal miR-500 Had Effect on Hepatic Stellate Cell Proliferation and Activation

**FIGURE 2:**
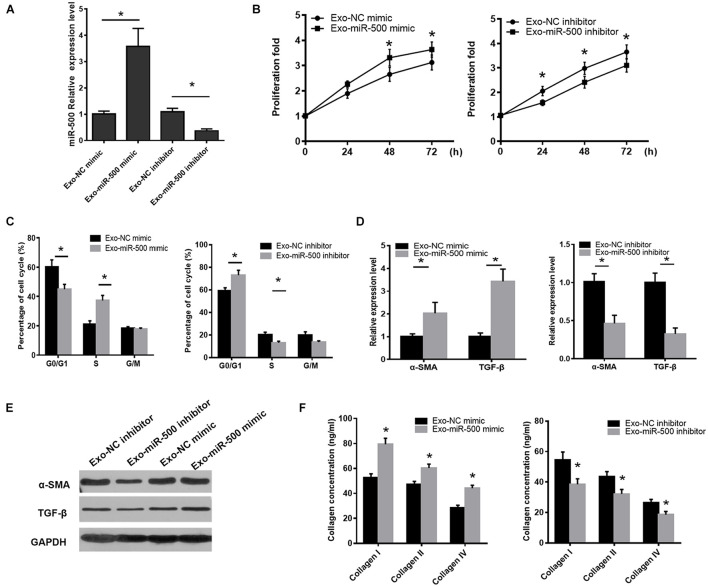
Exosomal miR-500 affects HSC proliferation and activation. **(A)** The expression levels of miR-500 in exosomes derived from macrophages transfected with miR-500 mimic or inhibitor and relative controls. **(B)** HSCs were treated with exosomes with miR-500 overexpression or inhibition, and then cell proliferation was determined by CCK-8 assays. **(C)** HSCs were treated with exosomes with miR-500 overexpression or inhibition, and the cell cycle was detected by using Flow cytometry. **(D)** HSCs were treated with exosomes with miR-500 overexpression or inhibition, and then the expression levels of α-SMA and TGF-β were detected by using qRT-PCR. **(E)** HSCs were treated with exosomes with miR-500 overexpression or inhibition, and then the expression levels of α-SMA and TGF-β were detected by using western blot. **(F)** HSCs were treated with exosomes with miR-500 overexpression or inhibition, and then the concentrations of type I, III, and IV collagen in the cell culture supernatant were tested by ELISA. The results are shown as mean ± SD of three independent experiments. *P < 0.05.

To define the functions of exosomal miR-500, we transfected miR-500 mimic or inhibitor or relative control sequences into the RAW264.7 cells, and then detected the change of exosomal miR-500 from the cells. The results are shown in Figure 2A. Next, we detected the effect of the exosomes with miR-500-overexpression or inhibition on the ability of cell proliferation and activation in HSCs. The results showed that the treatment of exosomes with miR-500 overexpression was able to promote the HSC proliferation, while the treatment of exosomes with miR-500 inhibition inhibited cell proliferation (Figure 2B). In addition, the treatment of exosomes with miR-500 inhibition induced G0–G1 cell cycle arrest; therefore, resulting in a significant decrease of cell percentage in the S-phase and considerable increase in G0/G1-phase. The treatment of exosomes with miR-500 overexpression showed opposite effect (Figure 2C). The treatment of exosomes with miR-500 overexpression also upregulated the mRNA and protein levels of α-SMA and TGF-β, while miR-500 inhibition could inhibit their levels (Figures 2D,E). We also showed that type I, type III, and type IV collagen were drastically increased in the culture supernatant of HSCs with the treatment of exosomes with miR-500 overexpression, while the group with the treatment of exosomes with miR-500 inhibition exerted opposite result (Figure 2F). These data indicated that exosomal miR-500 derived from macrophages might accelerate liver fibrosis.

**FIGURE 3:**
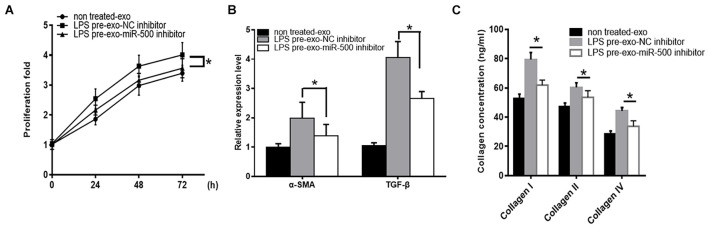
miR-500 inhibition suppresses LPS pre-exo-induced HSC proliferation and activation. **(A)** HSCs were treated with exosomes derived from LPS-treated macrophages with miR-500 inhibition or not, and then cell proliferation was detected by using CCK-8 assays. **(B)** HSCs were treated with exosomes derived from LPS-treated macrophages with miR-500 inhibition or not, and the expression levels of α-SMA and TGF-β were detected by qRT-PCR. **(C)** HSCs were treated with exosomes derived from LPS-treated macrophages with miR-500 inhibition or not, and the concentrations of type I, III, and IV collagen in the cell culture supernatant were tested by ELISA. The results are mean ± SD of three independent experiments. *P < 0.05.

To determine the importance of exosomal miR-500 in the crosstalk between LPS-stimulated macrophages and HSC, we inhibited the expression of exosome miR-500 in LPS-treated macrophages. As shown in Figure 3A, the treatment of exosomes derived from LPS-treated macrophages could promote HSC proliferation compared to non-treated cells, whereas the transfection of miR-500 inhibitor could rescue the LPS-induced upregulation of miR-500 and reverse the effects to a great extent. Meanwhile, the induction of α-SMA and TGF-β expression upon the treatment of exosomes from LPS-stimulated macrophages was partially suppressed by miR-500 inhibition (Figure 3B). Furthermore, type I, type III, and type IV collagen from HSCs with the treatment of exosomes from LPS-treated macrophages with miR-500 inhibition were significantly decreased compared to those without miR-500 inhibition (Figure 3C). Thus, these data suggested that exosomal miR-500 could promote HSC proliferation and activation, and it may play a vital important role in the crosstalk between macrophages and HSC.

### Exosomal miR-500 Accelerated Liver Fibrosis *in vivo*

**FIGURE 4:**
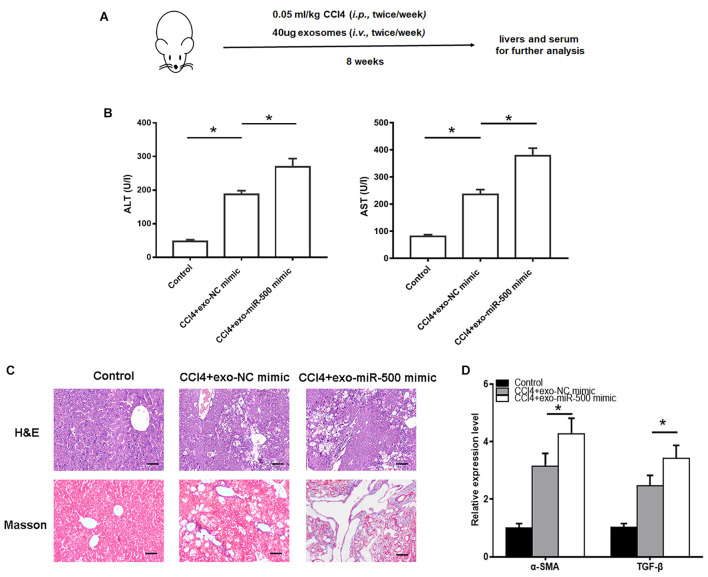
Exosomal miR-500 accelerates liver fibrosis *in vivo*. **(A)** The strategy for CCl_4_–induced liver fibrosis *in vivo*. **(B)** ALT and AST levels in mouse serum isolated from Control, CCl_4_+exo-NC mimic and CCl_4_+exo-miR-500 mimic groups were determined. Mean ± SD. *n* = 8. **(C)** H&E and Masson staining of mouse liver from Control, CCl_4_+exo-NC mimic and CCl_4_+exo-miR-500 mimic groups (magnification 100×). **(D)** The mRNA levels of α-SMA and TGF-β in liver tissues of different groups were assessed by qRT–PCR. *P < 0.05.

To further evaluate the roles of exosomal miR-500 in liver fibrosis *in vivo*, we used CCl_4_ to induce liver fibrosis mouse model, with the treatment of exosomes with miR-500 overexpression or not (Figure 4A). The treatment of 40 μg exosomes with miR-500 overexpression every week significantly accelerated liver injury induced by CCl_4_ treatment, compared with the group without miR-500 overexpression (Figure 4B). In addition, the H&E and Masson staining also showed that miR-500 overexpression accelerated liver fibrosis in live tissue slice (Figure 4C). The expression of some key genes involved in liver fibrosis, including α-SMA and TGF-β was found to be different in liver tissues from different groups (Figure 4D). The results showed that exosomal miR-500 accelerated liver fibrosis *in vivo*.

### Exosomal miR-500 Affected Hepatic Stellate Cell Proliferation and Activation and Accelerated Liver Fibrosis by Targeting MFN2

**FIGURE 5:**
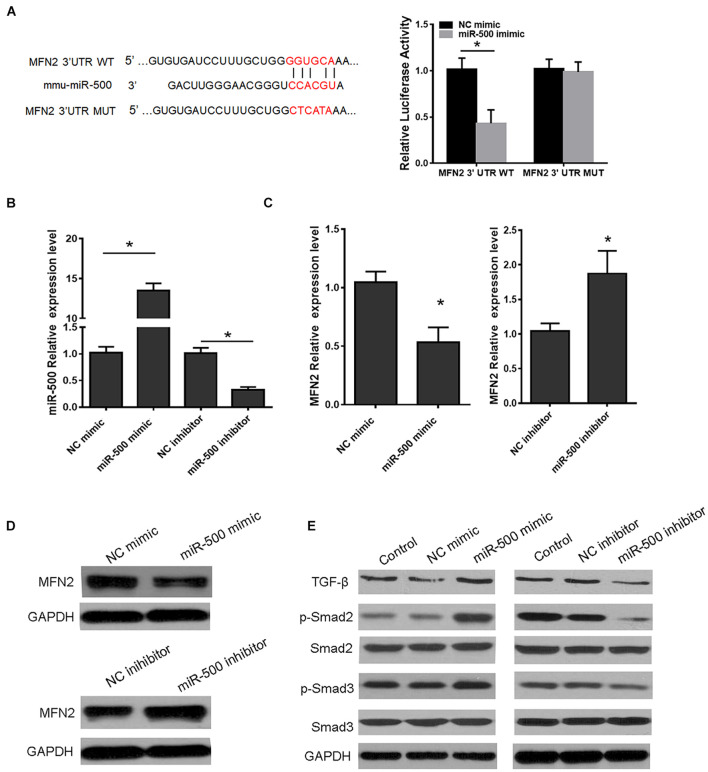
MFN2 is a target of miR-500. **(A)** Schema showing the binding sites of MFN2 3′ UTR with miR-500 (left) and luciferase reporter assays of HEK293 cells with transfection of pMIR-REPORT-WT/MUT MFN2 along with miR-500 mimic or NC mimic as indicated (right). **(B)** The expression level of miR-500 was detected in HSCs transfected with miR-500 mimic or inhibitor and relative controls. **(C)** The mRNA expression of MFN2 in HSCs transfected with miR-500 mimic or inhibitor were assessed by qRT-PCR. **(D)** The protein expression of MFN2 in HSCs with miR-500 overexpression or inhibition were determined by western blot. GAPDH was used as loading control. **(E)** The protein levels of TGF–β, p–Smad2/Smad2, and p–Smad3/Smad3, in HSCs with miR-500 overexpression or inhibition were assessed by western blot. *P < 0.05.

MFN2 has been demonstrated to be involved in many cellular processes including cell growth, apoptosis, autophagy, and ER stress. Of interest, by using TargetScan, MFN2 was predicted to be a putative target of miR-500 (Figure 5A). Relative luciferase activity of HEK293 cells co-transfected with WT or MUT MFN2 3′ UTR with miR-500 mimic or not showed that miR-500 mimic decreased MFN2 translation, whereas the luciferase activity of the cells transfected with Mut MFN2 3′ UTR was not affected by miR-500 mimic (Figure 5A). Then, we transfected miR-500 mimic or inhibitor into RAW264.7 cells, and determine the effects of miR-500 mimic or inhibitor on MFN2 expression. As shown in Figure 5B, the transfection of miR-500 mimic or inhibitor could upregulate or downregulate the expression of miR-500 in RAW264.7 cells. Furthermore, the mRNA and protein levels of MFN2 were significantly decreased in HSCs with miR-500 overexpression in comparison with control cells (Figures 5C,D and Supplementary Figure 1). In addition, miR-500 also resulted in changed levels of downstream targets of MFN2. The overexpression of miR-500 could promote the protein expression of TGF−β, and phosphorylated levels of Smad2/Smad3, while miR-500 inhibition had opposite effects (Figure 5E and Supplementary Figure 2).

**FIGURE 6:**
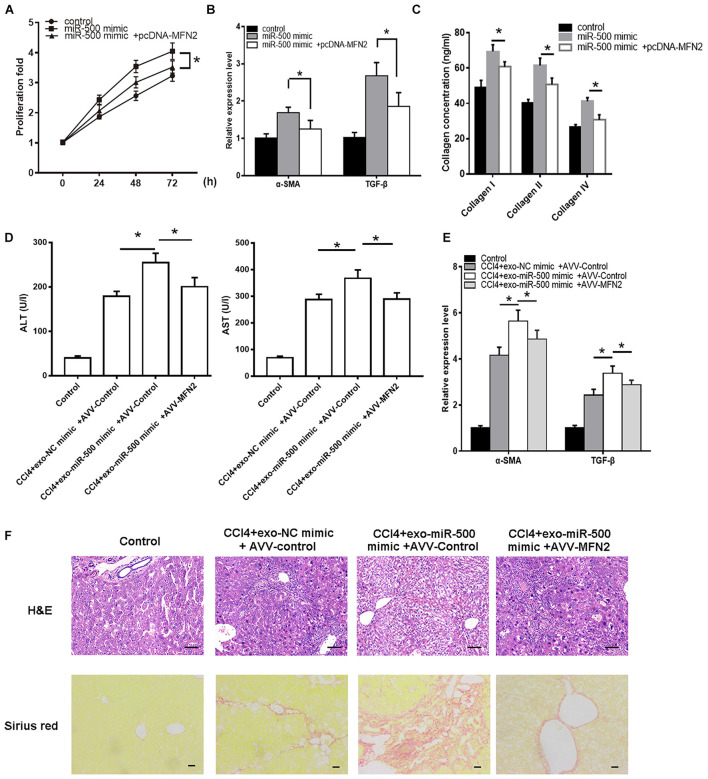
MFN2 reverses the effect of exosomal miR-500 on liver fibrosis *in vitro* and *in vivo*. **(A)** HSCs with transfection of pcDNA-MFN2 or not were treated with exosomes derived from LPS-treated macrophages with or without miR-500 overexpression, and then cell proliferation was determined using CCK-8 assays. **(B)** HSCs with transfection of pcDNA-MFN2 or not were treated with exosomes derived from LPS-treated macrophages with or without miR-500 overexpression, and the expression of α-SMA and TGF-β was detected by using qRT-PCR. **(C)** HSCs with transfection of pcDNA-MFN2 or not were treated with exosomes derived from LPS-treated macrophages with or without miR-500 overexpression, and the concentrations of type I, III, and IV collagen in cell culture supernatants were measured by ELISA. **(D)** ALT and AST levels in mouse serum from Control, CCl_4_+ exo-NC mimic+ AVV-Control, CCl_4_+exo-miR-500 mimic+ AVV-control, and CCl_4_+exo-miR-500 mimic+ AVV-MFN2 groups were determined. Mean ± SD. *n* = 8. **(E)** The expression of α-SMA and TGF-β in mouse liver from Control, CCl_4_+ exo-NC mimic+ AVV-Control, CCl_4_+exo-miR-500 mimic+ AVV-control, and CCl_4_+exo-miR-500 mimic+ AVV-MFN2 groups were determined by qRT-PCR. **(F)** H&E and Sirius Red staining of mouse liver from Control, CCl_4_+ exo-NC mimic+ AVV-Control, CCl_4_+exo-miR-500 mimic+ AVV-control, and CCl_4_+exo-miR-500 mimic+ AVV-MFN2 groups (magnification 100×). *P < 0.05.

We then determined whether miR-500-mediated MFN2 is involved in HSC activation. miR-500 mimic was transfected into HSCs with MFN2 overexpressing vector or control vector. We found that miR-500 mimic could significantly facilitate the HSC proliferation and activation, whereas the promotion was counteracted by the transfection of MFN2 overexpressing vector (Figures 6A–C). We also detected the interaction effects of exosomal miR-500 and MFN2 in liver fibrosis mouse model. The healthy C57/B6 mice were randomly divided into four groups, including control group, CCl_4_+exo-NC mimic +AVV-control group, CCl_4_+exo-miR-500 mimic+ AVV-control group, and CCl_4_+exo-miR-500+AVV-MFN2 group by tail injection. The results showed that the treatment of exosomes with miR-500 overexpression markedly increased the levels of ALT and AST, and the expression levels of α-SMA and TGF-β, while overexpression of MFN2 could inhibit the effect (Figures 6D,E). Furthermore, the H&E and Sirius Red staining also showed that exosomes with miR-500 overexpression could accelerate liver fibrosis, while MFN2 could partially inhibit it (Figure 6F).

These data suggested that MFN2 is a target of miR-500, and miR-500 could regulate MFN2 expression, therefore affecting the phosphorylated levels of Smad2/Smad3 and the protein levels of TGF-β. And miR-500 could accelerate liver fibrosis through MFN2 both *in vitro* and *in vivo*.

### The Expression Levels of miR-500 in Circulating Exosomes From Chronic Hepatic Disease Patients

**FIGURE 7:**
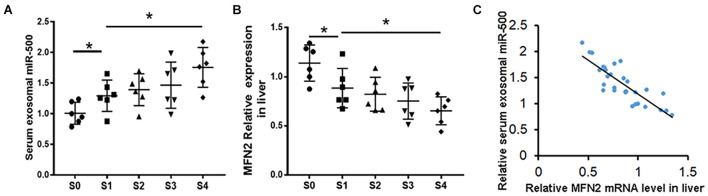
miR-500 in serum exosomes might be a potential biomarker for the development of liver fibrosis. **(A)** Relative expression of miR-500 in serum exosomes isolated from CHD patients including S0 (*n* = 6), S1 (*n* = 6), S2 (*n* = 6), S3 (*n* = 6), and S4 (*n* = 6). **(B)** The expression levels of MFN2 in liver tissues of CHD patients in different stages of liver fibrosis were detected by qRT-PCR. **(C)** Correlation analysis of exosomal miR-500 in serum and MFN2 in human liver tissues. Data are represented as mean ± SD of three replicates. *P < 0.05.

Next, circulating exosomes from 30 patients with CHD, fibrotic stage S0 (*n* = 6), S1 (*n* = 6), S2 (*n* = 6), S3 (*n* = 6), and S4 (*n* = 6) were isolated, and the levels of exosomal miR-500 were detected. As shown in Figure 7A, patients at the stage of S1–S4 have higher levels of exosomal miR-500 compared with patients at S0. Furthermore, serum exosomal miR-500 was higher in patients at S4 stage compared with those in early stages. We also determined the expression levels of MFN2 in liver tissues. The expression of MFN2 was decreased in the S1–S4 group (Figure 7B), and MFN2 expression level was gradually becoming lower in the development of liver fibrosis. Next, we explored the association of MFN2 expression in liver with exosomal miR-500. The data indicated that the mRNA expression of MFN2 in liver tissues was negatively correlated with serum exosomal miR-500 in CHD patients (Figure 7C).

## Discussion

Liver fibrosis is a highly debilitating pathology caused by continuous wound healing during chronic liver injury. HSCs act as a prominent player in the process of liver fibrosis because of its ability to produce fibrogenic proteins, α-SMA and CoL1A1 (Parsons et al., 2007; Friedman, 2008; Hernandez-Gea and Friedman, 2011; Parola and Pinzani, 2019).

Furthermore, it has been reported that the crosstalk between HSCs and immune cells in the local microenvironment also plays a crucial role in liver fibrosis. Of note, exosomes are recently reported as an important vesicle between cells, such as HSCs, damaged liver cells, liver macrophages and other immune cells. For example, exosomal miR-223 derived from NK cells could inhibit HSC activation by inhibiting autophagy (Wang L. et al., 2020); exosomal miR-199a-5p could suppress activated the HSC phenotype by targeting CCN2 (Chen et al., 2016); exosomes derived from miR-181-5p-modified adipose-derived mesenchymal stem cells prevent HSC activation *via* targeting STAT3 and Bcl2 (Qu et al., 2017); in addition, exosomal miR-214 from HSCs could be shuttled into both HSC and hepatic cells (Chen et al., 2014). Our previous study also showed that exosomal miR-103-3p derived from LPS-activated THP-1 macrophage is also participated in the HSC proliferation and activation (Chen et al., 2020). In this study, we showed that miR-500 was highly expressed in exosomes derived from LPS-treated macrophages and overexpression of miR-500 in macrophage exosomes significantly promoted HSC proliferation and activation, as proved by increased cell growth rate and production of α-SMA and TGF-β in HSCs, whereas exosomes derived from macrophages with miR-500 inhibition had opposite effects. Meanwhile, we also observed that the treatment of exosomes with miR-500 overexpression could accelerate the CCl_4_-induced liver fibrosis *in vivo*. Thus, these results revealed that exosomal miR-500 could promote the development of liver fibrosis.

miR-500 has high sequence identity among zebrafish, mice, and humans, indicating its evolutionarily conserved functions. miR-500 has been identified to be implicated in the tumorigenesis of multiple cancers. miR-500 could promote cell growth *via* targeting LRP1B in prostate cancer (Zhang et al., 2019). miR-500 promotes non-small cell lung cancer proliferation, migration, and invasion by targeting ING1 (Jiang et al., 2018). miR-500 has also been reported as a potential diagnostic biomarker for hepatocellular carcinoma (Yamamoto et al., 2009). miR-500 has also been found to be upregulated in cirrhotic livers (Vuppalanchi et al., 2013). However, its functions and the relative mechanisms in liver fibrosis have not been clarified yet. Herein, we demonstrated that miR-500 suppresses MFN2 expression in mouse HSCs *via* its direct targeting of MFN2 3′ UTR and that miR-500 could also affect the targets of MFN2 downstream, TGF−β/Smad signaling pathway. MFN2 belongs to the mitochondrial fusion protein (Mitofusin, MFN) family, which are required for mitochondrial outer membrane fusion (Rojo et al., 2002). MFN2 plays a vital important role in many cellular processes including cell proliferation, apoptosis, autophagy, and ER stress (Chen et al., 2012; Filadi et al., 2018). Zhu et al. (2020) previously demonstrated that MFN2 could promote HSC apoptosis and ameliorate liver fibrosis by regulating TGF−β/Smad signaling. Herein, our study emphasized the importance of MFN2 in liver fibrosis, since that MFN2 could strongly reverse the effect of exosomal miR-500 on liver fibrosis both *in vitro* and *in vivo*. Based on the study, our hypothesis was provided that exosomes containing upregulated miR-500 was secreted from LPS-activated macrophages and then was uptaken by HSCs, subsequently suppressing MFN2 in HSCs and leading to HSC activation and the development of liver fibrosis.

In addition, we determined the levels of serum exosomal miR-500 and the expression of MFN2 in liver fibrosis tissues. The results showed that miR-500 in serum exosomes from patients in S1–S4 stages was increased compared with those in S0 stage. And a significant difference between early and advanced stage liver fibrosis group was also observed. Therefore, circulating exosomes miR-500 could be function as a biomarker for the diagnosis of advanced liver fibrosis. Furthermore, the expression of MFN2 in liver tissues was lower in the S1–S4 group when compared with the S0 group. And the MFN2 expression in liver tissues was gradually becoming lower and lower during the process of liver fibrosis. And the mRNA expression levels of MFN2 in liver tissues were negatively correlated with serum exosomal miR-500 in CHD patients. These data suggested exosomal miR-500 and MFN2 as a putative biomarker for liver fibrosis.

**FIGURE 8:**
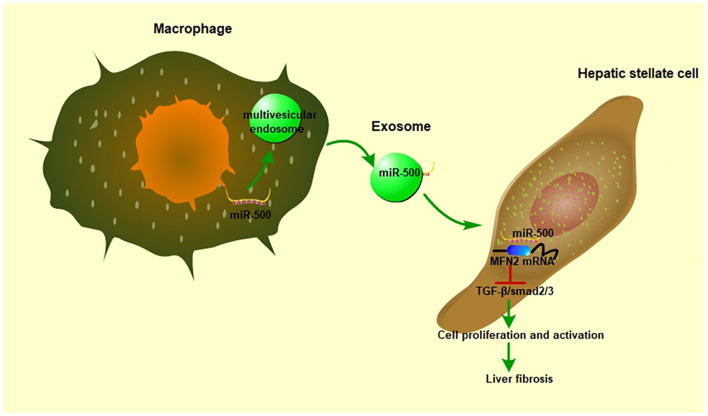
The role of exosomal miR-500 in the communication between macrophages and HSCs. In LPS-treated macrophages, miR-500 was highly expressed and then included into the multivesicular endosomes, which were secreted into intercellular space. After these exosomes being uptaken by recipient cells, the miR-500 in exosomes would inhibit the expression MFN2 by binding with its 3′ UTR, consequently promoting the proliferation and activation of HSC *via* TGF-β/Smad signaling pathway and accelerating liver fibrosis.

In conclusion, our study demonstrated that exosomal miR-500 derived from LPS-activated macrophages promoted HSC activation by suppressing TGF−β/Smad *via* targeting MFN2 (Figure 8). Exosomal miR-500 might be a potential biomarker for clinical diagnosis and a putative therapeutic strategy against liver fibrosis.

## Data Availability Statement

The raw data supporting the conclusions of this article will be made available by the authors, without undue reservation.

## Ethics Statement

The studies involving human participants were reviewed and approved by the Ethics Committee of the Second Xiangya Hospital of Central South University. The patients/participants provided their written informed consent to participate in this study. The animal study was reviewed and approved by the Ethics Committee of the Second Xiangya Hospital of Central South University.

## Author Contributions

XL was responsible for administrating the study and writing this manuscript. LC and YH were responsible for doing the experiments. ZD and PH were responsible for analyzing the data. HY was responsible for helping to directing the processes of some experiments. YZ and QJ were responsible for sample collection. All authors contributed to the article and approved the submitted version.

## Conflict of Interest

The authors declare that the research was conducted in the absence of any commercial or financial relationships that could be construed as a potential conflict of interest.

## Publisher’s Note

All claims expressed in this article are solely those of the authors and do not necessarily represent those of their affiliated organizations, or those of the publisher, the editors and the reviewers. Any product that may be evaluated in this article, or claim that may be made by its manufacturer, is not guaranteed or endorsed by the publisher.

## Supplementary Material

Supplementary Figure 1 The western blot quantitation for Figure 5E.

Supplementary Figure 2 The western blot quantitation for Figure 5F.

## References

[B1] Bartel D. P. (2004). MicroRNAs: genomics, biogenesis, mechanism, and function. *Cell* 116 281–297. 10.1016/s0092-8674(04)00045-5 14744438

[B2] Bataller R. Brenner D. A. (2005). Liver fibrosis. *J. Clin. Invest.* 115 209–218. 10.1172/JCI24282 15690074 PMC546435

[B3] Bhogal R. K. Bona C. A. (2005). B cells: no longer bystanders in liver fibrosis. *J. Clin. Invest.* 115 2962–2965. 10.1172/JCI26845 16276407 PMC1265880

[B4] Casini A. Ricci O. E. Paoletti F. Surrenti C. (1985). Immune mechanisms for hepatic fibrogenesis. T-lymphocyte-mediated stimulation of fibroblast collagen production in chronic active hepatitis. *Liver* 5 134–141. 10.1111/j.1600-0676.1985.tb00228.x 3876501

[B5] Chen L. Charrier A. Zhou Y. Chen R. Yu B. Agarwal K. (2014). Epigenetic regulation of connective tissue growth factor by MicroRNA-214 delivery in exosomes from mouse or human hepatic stellate cells. *Hepatology* 59 1118–1129. 10.1002/hep.26768 24122827 PMC3943742

[B6] Chen L. Chen R. Velazquez V. M. Brigstock D. R. (2016). Fibrogenic Signaling Is Suppressed in Hepatic Stellate Cells through Targeting of Connective Tissue Growth Factor (CCN2) by Cellular or Exosomal MicroRNA-199a-5p. *Am. J. Pathol.* 186 2921–2933. 10.1016/j.ajpath.2016.07.011 27662798 PMC5222964

[B7] Chen L. Yao X. Yao H. Ji Q. Ding G. Liu X. (2020). Exosomal miR-103-3p from LPS-activated THP-1 macrophage contributes to the activation of hepatic stellate cells. *FASEB J.* 34 5178–5192. 10.1096/fj.201902307RRR 32061112

[B8] Chen Y. Csordas G. Jowdy C. Schneider T. G. Csordas N. Wang W. (2012). Mitofusin 2-containing mitochondrial-reticular microdomains direct rapid cardiomyocyte bioenergetic responses via interorganelle Ca(2+) crosstalk. *Circ. Res.* 111 863–875. 10.1161/CIRCRESAHA.112.266585 22777004 PMC3444672

[B9] Feili X. Wu S. Ye W. Tu J. Lou L. (2018). MicroRNA-34a-5p inhibits liver fibrosis by regulating TGF-beta1/Smad3 pathway in hepatic stellate cells. *Cell Biol. Int.* 42 1370–1376. 10.1002/cbin.11022 29957876

[B10] Filadi R. Pendin D. Pizzo P. (2018). Mitofusin 2: from functions to disease. *Cell Death Dis.* 9:330. 10.1038/s41419-017-0023-6 29491355 PMC5832425

[B11] Fouts D. E. Torralba M. Nelson K. E. Brenner D. A. Schnabl B. (2012). Bacterial translocation and changes in the intestinal microbiome in mouse models of liver disease. *J. Hepatol.* 56 1283–1292. 10.1016/j.jhep.2012.01.019 22326468 PMC3357486

[B12] Friedman S. L. (2008). Mechanisms of hepatic fibrogenesis. *Gastroenterology* 134 1655–1669. 10.1053/j.gastro.2008.03.003 18471545 PMC2888539

[B13] Glassner A. Eisenhardt M. Kokordelis P. Kramer B. Wolter F. Nischalke H. D. (2013). Impaired CD4(+) T cell stimulation of NK cell anti-fibrotic activity may contribute to accelerated liver fibrosis progression in HIV/HCV patients. *J. Hepatol.* 59 427–433. 10.1016/j.jhep.2013.04.029 23665286

[B14] Gupta P. Sata T. N. Yadav A. K. Mishra A. Vats N. Hossain M. M. (2019). TGF-beta induces liver fibrosis via miRNA-181a-mediated down regulation of augmenter of liver regeneration in hepatic stellate cells. *PLoS One* 14:e0214534. 10.1371/journal.pone.0214534 31166951 PMC6550375

[B15] Hernandez-Gea V. Friedman S. L. (2011). Pathogenesis of liver fibrosis. *Annu. Rev. Pathol.* 6 425–456. 10.1146/annurev-pathol-011110-130246 21073339

[B16] Heymann F. Trautwein C. Tacke F. (2009). Monocytes and macrophages as cellular targets in liver fibrosis. *Inflamm. Allergy Drug Targets* 8 307–318. 10.2174/187152809789352230 19534673

[B17] Jiang M. Zhou L. Y. Xu N. An Q. (2018). Down-regulation of miR-500 and miR-628 suppress non-small cell lung cancer proliferation, migration and invasion by targeting ING1. *Biomed. Pharmacother.* 108 1628–1639. 10.1016/j.biopha.2018.09.145 30372865

[B18] Johnstone R. M. (2006). Exosomes biological significance: a concise review. *Blood Cell Mol. Dis.* 36 315–321. 10.1016/j.bcmd.2005.12.001 16487731

[B19] Lawrence T. Natoli G. (2011). Transcriptional regulation of macrophage polarization: enabling diversity with identity. *Nat. Rev. Immunol.* 11 750–761. 10.1038/nri3088 22025054

[B20] Li H. You H. Fan X. Jia J. (2016). Hepatic macrophages in liver fibrosis: pathogenesis and potential therapeutic targets. *BMJ Open Gastroenterol.* 3:e000079. 10.1136/bmjgast-2016-000079 27252881 PMC4885270

[B21] Liu M. Hu Y. Yuan Y. Tian Z. Zhang C. (2019). gammadeltaT cells suppress liver fibrosis via strong cytolysis and enhanced NK cell-mediated cytotoxicity against hepatic stellate cells. *Front. Immunol.* 10:477. 10.3389/fimmu.2019.00477 30930903 PMC6428727

[B22] Parola M. Pinzani M. (2019). Liver fibrosis: pathophysiology, pathogenetic targets and clinical issues. *Mol. Aspects Med.* 65 37–55. 10.1016/j.mam.2018.09.002 30213667

[B23] Parsons C. J. Takashima M. Rippe R. A. (2007). Molecular mechanisms of hepatic fibrogenesis. *J. Gastroenterol. Hepatol.* 22(Suppl. 1) S79–S84. 10.1111/j.1440-1746.2006.04659.x 17567474

[B24] Pradere J. P. Kluwe J. De Minicis S. Jiao J. J. Gwak G. Y. Dapito D. H. (2013). Hepatic macrophages but not dendritic cells contribute to liver fibrosis by promoting the survival of activated hepatic stellate cells in mice. *Hepatology* 58 1461–1473. 10.1002/hep.26429 23553591 PMC3848418

[B25] Qu Y. Zhang Q. Cai X. Li F. Ma Z. Xu M. (2017). Exosomes derived from miR-181-5p-modified adipose-derived mesenchymal stem cells prevent liver fibrosis via autophagy activation. *J. Cell. Mol. Med.* 21 2491–2502. 10.1111/jcmm.13170 28382720 PMC5618698

[B26] Ramachandran P. Iredale J. P. (2012). Macrophages: central regulators of hepatic fibrogenesis and fibrosis resolution. *J. Hepatol.* 56 1417–1419. 10.1016/j.jhep.2011.10.026 22314426

[B27] Rojo M. Legros F. Chateau D. Lombes A. (2002). Membrane topology and mitochondrial targeting of mitofusins, ubiquitous mammalian homologs of the transmembrane GTPase Fzo. *J. Cell. Sci.* 115(Pt. 8) 1663–1674. 11950885 10.1242/jcs.115.8.1663

[B28] Tacke F. Zimmermann H. W. (2014). Macrophage heterogeneity in liver injury and fibrosis. *J. Hepatol.* 60 1090–1096. 10.1016/j.jhep.2013.12.025 24412603

[B29] Thery C. Zitvogel L. Amigorena S. (2002). Exosomes: composition, biogenesis and function. *Nat. Rev. Immunol.* 2 569–579. 10.1038/nri855 12154376

[B30] van Niel G. Porto-Carreiro I. Simoes S. Raposo G. (2006). Exosomes: a common pathway for a specialized function. *J. Biochem.* 140 13–21. 10.1093/jb/mvj128 16877764

[B31] Vuppalanchi R. Liang T. Goswami C. P. Nalamasu R. Li L. Jones D. (2013). Relationship between differential hepatic microRNA expression and decreased hepatic cytochrome P450 3A activity in cirrhosis. *PLoS One* 8:e74471. 10.1371/journal.pone.0074471 24058572 PMC3772944

[B32] Wallace M. C. Friedman S. L. Mann D. A. (2015). Emerging and disease-specific mechanisms of hepatic stellate cell activation. *Semin. Liver Dis.* 35 107–118. 10.1055/s-0035-1550060 25974897

[B33] Wang L. Wang Y. Quan J. (2020). Exosomal miR-223 derived from natural killer cells inhibits hepatic stellate cell activation by suppressing autophagy. *Mol. Med.* 26:81. 10.1186/s10020-020-00207-w 32873229 PMC7465359

[B34] Wang Q. Wei S. Zhou H. Li L. Zhou S. Shi C. (2020). MicroRNA-98 inhibits hepatic stellate cell activation and attenuates liver fibrosis by regulating HLF expression. *Front. Cell Dev. Biol.* 8:513. 10.3389/fcell.2020.00513 32637414 PMC7316892

[B35] Xuan J. Guo S. L. Huang A. Xu H. B. Shao M. Yang Y. (2017). MiR-29a and miR-652 attenuate liver fibrosis by inhibiting the differentiation of CD4+ T Cells. *Cell Struct. Funct.* 42 95–103. 10.1247/csf.17005 28768954

[B36] Yamamoto Y. Kosaka N. Tanaka M. Koizumi F. Kanai Y. Mizutani T. (2009). MicroRNA-500 as a potential diagnostic marker for hepatocellular carcinoma. *Biomarkers* 14 529–538. 10.3109/13547500903150771 19863192

[B37] Yin C. Evason K. J. Asahina K. Stainier D. Y. (2013). Hepatic stellate cells in liver development, regeneration, and cancer. *J. Clin. Invest.* 123 1902–1910. 10.1172/JCI66369 23635788 PMC3635734

[B38] Zhang Z. Cui R. Li H. Li J. (2019). miR-500 promotes cell proliferation by directly targetting LRP1B in prostate cancer. *Biosci. Rep.* 39:BSR20181854. 10.1042/BSR20181854 30877185 PMC6449515

[B39] Zhu H. Shan Y. Ge K. Lu J. Kong W. Jia C. (2020). Specific overexpression of mitofusin-2 in hepatic stellate cells ameliorates liver fibrosis in mice model. *Hum. Gene Ther.* 31 103–109. 10.1089/hum.2019.153 31802713

