# Supplementary figures and images for: Exosomal miR-500 Derived From Lipopolysaccharide-Treated Macrophage Accelerates Liver Fibrosis by Suppressing MFN2
Source: Front Cell Dev Biol. 2021 Oct 5;9:716209. doi: 10.3389/fcell.2021.716209 (PMC8525629; doi:10.3389/fcell.2021.716209)

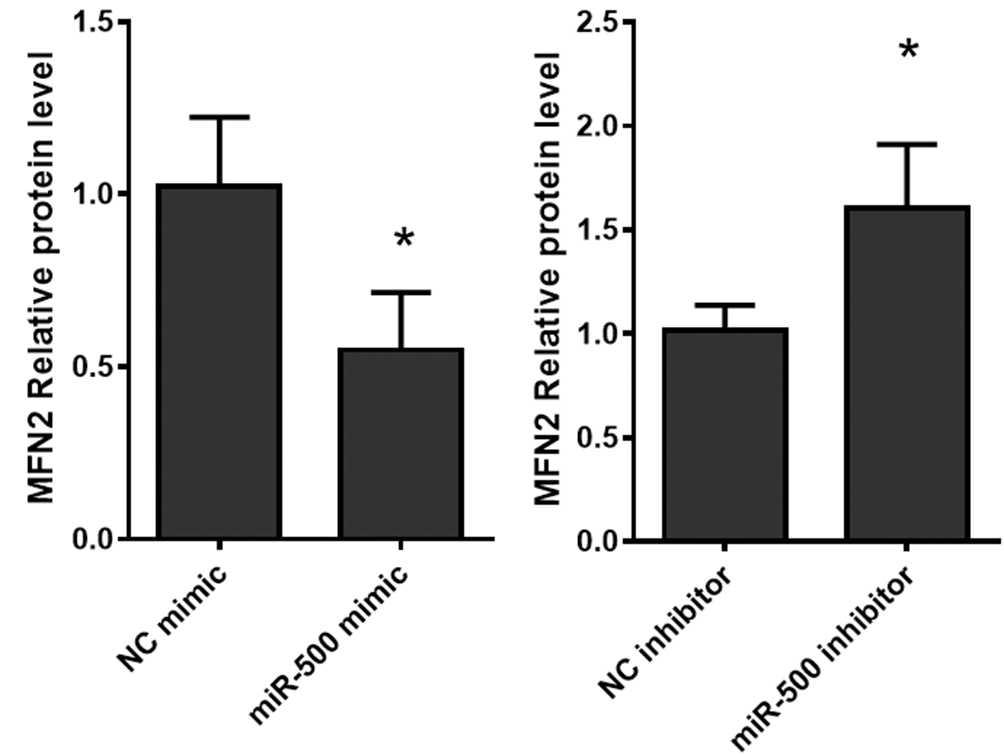

Supplement: Supplementary Figure 1 — The western blot quantitation for Figure 5E. [file Image_1.TIF]

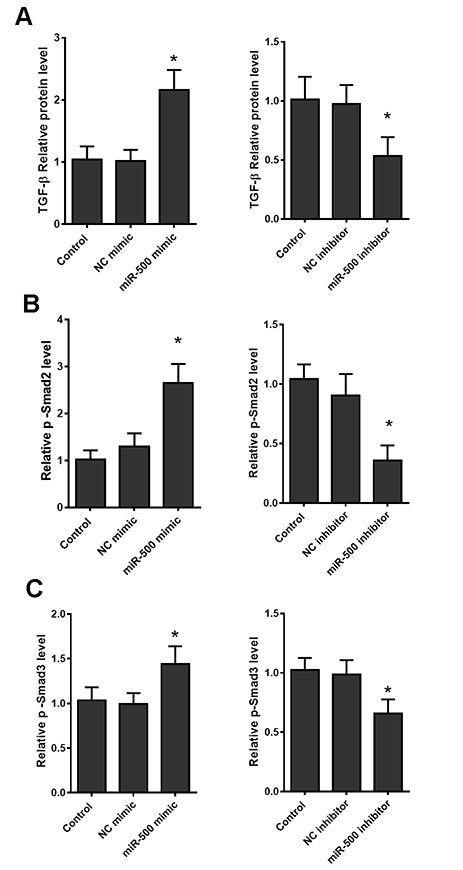

Supplement: Supplementary Figure 2 — The western blot quantitation for Figure 5F. [file Image_2.TIF]
